# Impact of the material and sintering protocol, layer thickness, and thermomechanical aging on the two-body wear and fracture load of 4Y-TZP crowns

**DOI:** 10.1007/s00784-022-04616-5

**Published:** 2022-07-15

**Authors:** Felicitas Mayinger, Ramona Buser, Maximilian Laier, Lisa Marie Schönhoff, Matthias Kelch, Rüdiger Hampe, Bogna Stawarczyk

**Affiliations:** 1 Department of Prosthetic Dentistry, Dental School, LMU Munich, Goethestraße 70, 80336 Munich, Germany; 2 Department of Reconstructive Dentistry and Gerodontology, University of Bern, Freiburgstrasse 7, 3007 Bern, Switzerland

**Keywords:** Zirconia, Fracture load, Two-body wear, High-speed sintering, Layer thickness

## Abstract

**Objectives:**

The aim of this study is to investigate the influence of the material and corresponding sintering protocol, layer thickness, and aging on the two-body wear (2BW) and fracture load (FL) of 4Y-TZP crowns.

**Materials and methods:**

Multi-layer 4Y-TZP crowns in three thicknesses (0.5 mm/1.0 mm/1.5 mm) were sintered by high-speed (Zolid RS) or conventional (Zolid Gen-X) sintering. 2BW of ceramic and enamel antagonist after aging (1,200,000 mechanical-, 6000 thermal-cycles) was determined by 3D-scanning before and after aging and subsequent matching to determine volume and height loss (6 subgroups, *n* = 16/subgroup). FL was examined initially and after aging (12 subgroups, *n* = 16/subgroup). Fractographic analyses were performed using light-microscope imaging. Global univariate analysis of variance, one-way ANOVA, linear regression, Spearman’s correlation, Kolgomorov–Smirnov, Mann–Whitney *U*, and *t* test were computed (alpha = 0.05). Weibull moduli were determined. Fracture types were analyzed using Ciba Geigy table.

**Results:**

Material/sintering protocol did not influence 2BW (crowns: *p* = 0.908, antagonists: *p* = 0.059). High-speed sintered Zolid RS presented similar (*p* = 0.325–0.633) or reduced (*p* < 0.001–0.047) FL as Zolid Gen-X. Both 4Y-TZPs showed an increased FL with an increasing thickness (0.5(797.3–1429 N) < 1.0(2087–2634 N) < 1.5(2683–3715 N)mm; *p* < 0.001). For most groups, aging negatively impacted FL (*p* < 0.001–0.002). Five 0.5 mm specimens fractured, four showed cracks during and after aging.

**Conclusions:**

High-speed sintered crowns with a minimum thickness of 1.0 mm showed sufficient mechanical properties to withstand masticatory forces, even after a simulated aging period of 5 years.

**Clinical relevance:**

Despite the manufacturer indicating a thickness of 0.5 mm to be suitable for single crowns, a minimum thickness of 1.0 mm should be used to ensure long-term satisfactory results.

## Introduction

Due to their high biocompatibility and excellent processability that ensures a precise marginal fit, ceramics represent the material of choice for esthetic tooth-colored prosthetic restorations [1–3]. Zirconia ceramics can withstand the masticatory forces occurring in the posterior region and furthermore allow for the manufacturing of multi-unit fixed dental prostheses [4]. As of today, 5 zirconia generations are available. These differ in their yttrium oxide content and therefore, their tetragonal and cubic phase content and their optical and mechanical properties. The high-strength 3 mol% yttria stabilized-tetragonal zirconia polycrystal (3Y-TZP) typically consists of ∼80 wt% tetragonal and ∼20 wt% cubic zirconia; the “all-rounder” 4Y-TZP contains ∼70 wt% tetragonal and 30 wt% cubic zirconia [5]. Theoretically, 3Y-TZP is stabilized in the tetragonal phase and can undergo tetragonal to monoclinic phase transformations [6], whereas an increase in the yttrium oxide content and in consequence of the cubic phase yields fully stabilized zirconia materials [7, 8]. In the workflow of lithium silicate ceramics, the treatment with a prosthetic restoration that is manufactured in one single appointment is firmly established [9]. Against this background, the time-consuming sintering of zirconia with conventional sintering protocols used to limit the applicability of this material group and led to the development of speed- and high-speed sintering protocols. These allow a shortening of the sintering time from nearly 10 h to a minimum of 10 min. In a sinter temperature–sinter duration graph, the area under the sinter curve (AUC) above a sinter temperature of 1200 °C correlates with the mechanical and optical properties of the zirconia [10]. For 3Y-TZP, an increase in the AUC can increase the grain size and hereby lead to a decrease in the mechanical properties of the material, while simultaneously improving the esthetic appearance of the restoration [10–17]. High-speed sintering protocols were developed on the condition of maintaining the outstanding feature of zirconia, its mechanical properties. In consequence, blanks destined for high-speed sintering require a new coloring that anticipates the reduced AUC employed for high-speed sintering and the resultant loss of translucency [13]. This concept is incorporated in the workings of the Ceramill Therm RS, one of the two high-speed sintering furnaces that are available as of today. In this system, high-speed sintering is coupled with the use of Zolid RS, a multilayer 4Y-TZP. Its “conventional” counterpart is Zolid Gen-X. According to the manufacturer, the only difference between the two 4Y-TZP materials is a varying doting with pigments. First investigations have reported promising findings after high-speed sintering with an experimental furnace [13, 18–20]. The purpose of the present examination was to determine if those observations persist for the now commercially available high-speed sintering furnace Ceramill Therm RS and the related Zolid RS. Particular attention was paid to the question whether high-speed sintered crowns deliver sufficiently high mechanical properties in reduced layer thicknesses and after a prolonged time in situ, mimicked by thermomechanical aging with 1,200,000 chewing and 6000 thermal cycles, that are supposed to imitate a clinical situation after 5 years in vivo [21].

The aim of this investigation was to examine the influence of the material and corresponding sintering protocol, the restoration’s layer thickness, and thermomechanical aging in a chewing simulator on the two-body wear (2BW) and fracture load (FL) of 4Y-TZP crowns. The first hypothesis stated that the choice of material/sintering protocol does not present an impact on the 2BW of the ceramic crown or respective enamel antagonist. The second hypothesis stated that neither the material/sintering protocol nor the choice of three different layer thicknesses nor artificial aging show an impact on the fracture load of the 4Y-TZP crowns.

## Materials and methods

**Fig. 1:**
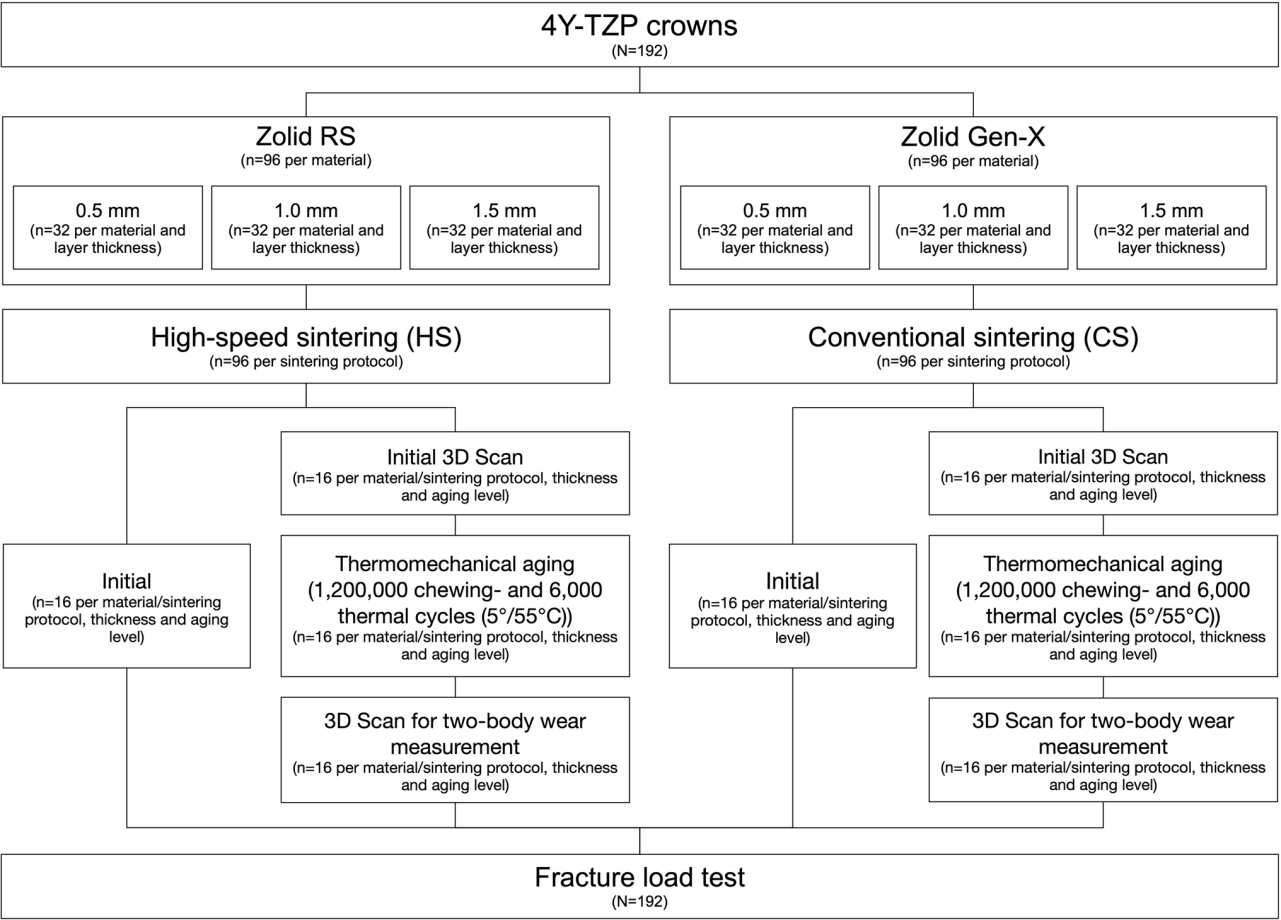
Study design

The 2BW and FL of two different multi-layer 4Y-TZP (Zolid RS (Lot No: 20191127-PR) and Zolid Gen-X (Lot No: 1912000), Amann Girrbach, Koblach, Austria) crowns manufactured in three layer thicknesses and sintered using either a high-speed (Zolid RS (HS)) or a conventional (Zolid Gen-X (CS)) sintering protocol were examined initially or after thermomechanical aging (Fig. 1).

### Specimen preparation

**Fig. 2:**
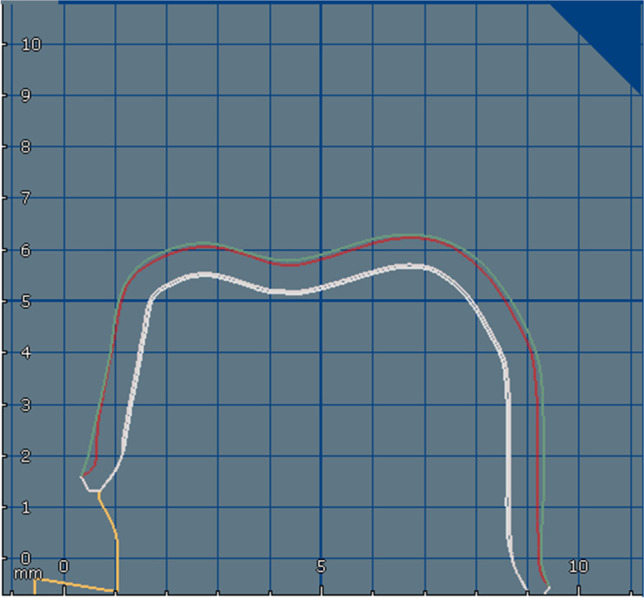
Design of the 0.5-mm-thick crown on the prepared maxillary first molar

The crowns for a maxillary first molar (tooth 16) were designed in three different occlusal layer thicknesses (0.5 mm (Fig. 2), 1.0 mm, 1.5 mm) using a CAD/CAM software (Ceramill Mind and Ceramill Match 2, Amann Girrbach) and manufactured with a milling machine (Ceramill Motion 2, Amann Girrbach). The milled crowns were detached from the presintered zirconia blanks using a diamond bur (Komet Dental, Lemgo, Germany). Subsequently, each specimen was meticulously cleaned to remove any remaining milling dust. Following the manufacturer’s instructions, Zolid RS crowns were sintered at 1580 °C (Ceramill Therm RS, Amann Girrbach), while Zolid Gen-X crowns were sintered at 1450 °C (Ceramill Therm 2, Amann Girrbach). The occlusal surface of each crown was polished with a ceramic polisher (Komet Dental) followed by a goat hair brush (Komet Dental) applied with a diamond polishing paste (Yeti Dia Glace, Yeti Dentalprodukte, Engen, Germany). The crowns were then bonded to the abutments (maxillary first molars with a height of 6.3 mm, a 360° chamfer preparation, and a taper of 10°) milled from fiberglass reinforced resin (TRINIA, Bicon, Boston, USA) using SoloCem (Coltène/Whaledent, Altstätten, Switzerland). The luting material was carefully applied into the inner surface of each crown and distributed equally with a micro brush before the crown was bonded to the abutment. Surplus luting material was carefully removed before polymerization was performed with Elipar DeepCure (3 M, Seefeld, Germany) for 20 s from the buccal, occlusal, and lingual side. Subsequently, all specimens were stored for 24 h in 37 °C tempered deionized water (HERAcell 150, Thermo Fisher Scientific, Waltham, USA).

### Thermomechanical aging

Half of the specimens were aged in a chewing simulator (Chewing Simulator CS-4.10, SD Mechatronik), resulting in the formation of 12 subgroups (*n* = 16 per material/sintering protocol, thickness, and aging level). Standardized enamel antagonists were obtained by separating the mesiobuccal cusps from maxillary human molars with a cut-off wheel (Komet Dental). The cusps were mounted in specimen holders for chewing simulation using amalgam (Dispersalloy, Dentsply Sirona, Konstanz, Germany). The tip of the cusps were then carefully adjusted to a spherical shape with a 40 µm bur (Komet Dental). The use of human teeth was approved by the responsible ethics committee. Specimens were loaded with 50 N for 1,200,000 mechanical cycles at 1.3 Hz. A sliding movement of 0.7 mm from the central fissure toward the buccal cusps was performed for each mechanical cycle. Simultaneously, specimens underwent 6000 thermal cycles in distilled water tempered to 5 °C/55 °C with a dwelling time of 60 s [18].

### Fracture types and fractographic analysis

Specimens were classified as intact, cracked, or fractured. Specimens presenting a crack were considered as survivals and thereafter tested for 2BW and FL, whereas specimens that fractured during thermomechanical aging were considered as non-survivals. A fractographic analysis was performed for all specimens that cracked or fractured during thermomechanical aging following the ADM guidelines [22, 23]. For this purpose, images were taken at a 20 × –500 × magnification using a digital light microscope with a large depth of field and a long observation distance (Keyence VHX-970F, Keyence, Osaka, Japan).

### Two-body wear measurement

To determine the material loss caused by thermomechanical aging, the occlusal surfaces of the zirconia crowns and respective enamel antagonists were scanned (LAS-20D, SD Mechatronik GmbH, Feldkirchen-Westerham, Germany) following the application of a scanning powder at a distance of 10 cm (Arti-Spray, white, BK 285, Dr. Jean Bausch, Köln, Germany). The three-dimensional scans were performed prior to (prescan) and after thermomechanical aging (postscan). The obtained scan data (pre- and postscan) were superimposed with the best fit method to calculate the volume and height loss from 2BW with a three-dimensional data measuring software (GOM Inspect, GOM, Braunschweig, Germany) [24].

### Fracture load test

**Fig. 3:**
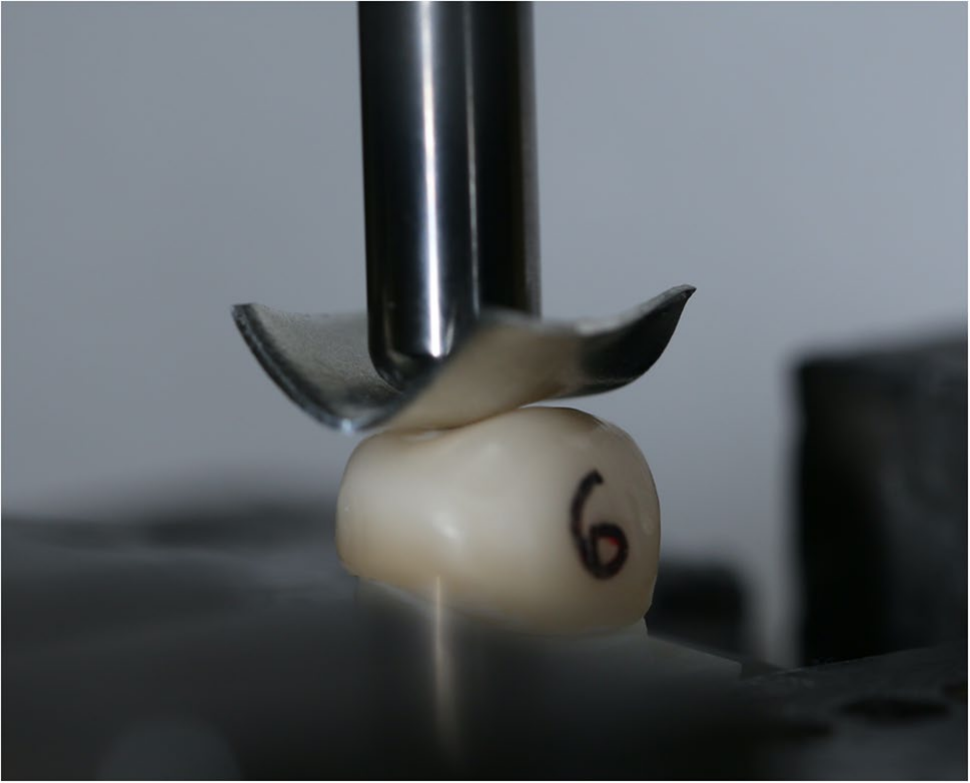
A Zolid RS/HS specimen (1.0 mm, initial) mounted for the fracture load test

FL was determined with the universal testing machine (Zwick 1445, ZwickRoell, Ulm, Germany) equipped with a chrome-nickel steel testing stamp (6 mm in diameter; SD Mechatronik) operating at a crosshead speed of 1 mm/min (Fig. 3). The stamp was carefully positioned on the occlusal surface of each crown. A 0.1 mm tin foil (Dentaurum, Ispringen, Germany) was placed between each specimen and the testing stamp to avoid force peaks. The FL measurement was stopped as soon as the maximum FL decreased by 10% [18].

### Statistical analysis

The sample size of *n* = 16 per subgroup was based on similar investigations, that observed significant differences between groups for similar or smaller sample sizes [18, 25, 26]. Data were descriptively analyzed and the Kolmogorov–Smirnov test was computed to test for a violation of the normal distribution. A linear regression was applied to disclose associations between the examined variables. Spearman’s correlation was calculated to test for a correlation between the volume and height values regarding 2BW. For 2BW, significant differences between the different groups were analyzed with the Mann–Whitney *U* test. The effect of the different variables on the FL was examined with global univariate analysis of variance, *t* test, and one-way ANOVA. Weibull moduli were determined with the maximum likelihood estimation method at a 95% confidence level [27]. The confidence intervals for the relative frequency of the different fracture types were determined using the Ciba Geigy table (95% confidence interval, set to (1–2α) × 100%). With the help of the confidence interval, the significant differences between the groups can be recognized purely descriptively [28–30]. *P* values below 0.05 were regarded as statistically significant (IBM Statistics SPSS 26.0, IBM, Amonk, USA).

## Results

### Two-body wear

Seventy-five percent of the tested 2BW groups showed a deviation from the normal distribution and were therefore analyzed non-parametrically. As a high correlation was observed between the volume and the height loss following 2BW (*p* < 0.001; crowns: *r* = 0.999, antagonists: *r* = 0.820), the parameter volume is displayed and discussed in the present manuscript and is hereafter referred to as material loss. The choice of material/sintering protocol did not impact the observed 2BW values (crowns: *p* = 0.908, antagonists: *p* = 0.059). The material loss of the enamel antagonist was higher than that reported for the crowns (*p* < 0.001) (Table 1).

**Table 1:** Descriptive statistics for the two-body wear [mm^3^] of the 4Y-TZP crowns and respective enamel antagonists

	Zolid RS/HS		Zolid Gen-X/CS	
	Mean ± SD	[95% CI]	Mean (± SD)	[95% CI]
4Y-TZP	− 0.01516 ± 0.0773	[− 0.0434; 0.0133]	− 0.00094 ± 0.0053	[− 0.0027; 0.0010]
Antagonist	− 0.60387 ± 0.3859	[− 0.7453; − 0.4624]	− 0.71750 ± 0.3355	[− 0.8383; − 0.5966]

### Fracture load and Weibull modulus

**Fig. 4:**
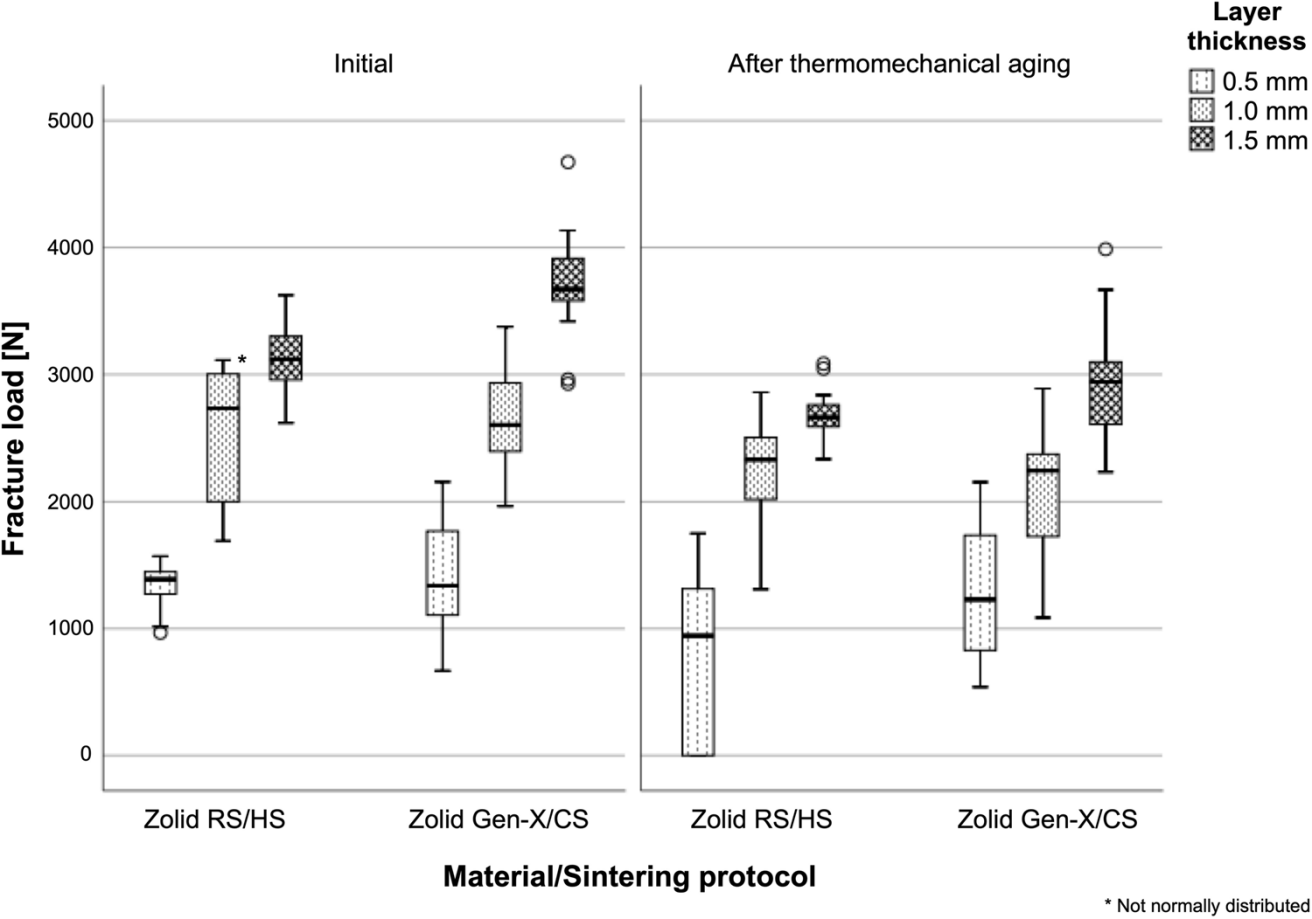
Fracture load values [N] for all tested groups

Only 8% of the FL groups showed a deviation from the normal distribution and were hence evaluated parametrically. The restorations’ layer thickness showed the highest impact on the FL (partial eta squared (ηp^2^) = 0.776, *p* < 0.001), followed by the aging level (ηp^2^ = 0.230, *p* < 0.001), and the combination of zirconia material and sintering protocol (ηp^2^ = 0.066, *p* < 0.001). The interaction between the layer thickness, the aging level, and the material/sintering protocol also affected the results (ηp^2^ = 0.038, *p* = 0.031). As the higher order interactions were found to be significant, the fixed effects of the tested parameters could not be directly compared. The linear regression showed an association between the examined variables (R^2^ = 0.759, *p* < 0.001) (Table 2). Consequently, the data were analyzed separately according to the tested hypotheses. Within the aged 0.5 mm crowns (*p* = 0.022) and all 1.5 mm crowns (initial: *p* < 0.001, after thermomechanical aging: *p* = 0.047), Zolid Gen-X/CS specimens presented higher FL values than observed for Zolid RS/HS (Fig. 4, Table 3). Zolid Gen-X/CS specimens showed a higher Weibull modulus than observed for Zolid RS/HS within the aged 0.5 mm crowns, while Zolid RS/HS presented higher Weibull moduli within the initial 0.5 mm and the aged 1.5 mm crowns. Both Zolid RS/HS and Gen-X/CS crowns showed an increase in their FL values with an increasing layer thickness (0.5 < 1.0 < 1.5 mm) (*p* < 0.001). For most groups, an increase in layer thickness resulted in an increased Weibull modulus. Within Zolid RS/HS (0.5 mm: *p* = 0.002, 1.5 mm: *p* < 0.001) and Gen-X/CS (1.0 mm: *p* = 0.002, 1.5 mm: *p* < 0.001) groups, thermomechanical aging showed a negative impact on the reported FL values. In the 0.5 mm Zolid RS/HS group, aging led to a decreased Weibull modulus.

**Table 2:** Estimates of the regression coefficients for fracture load

Variable	Unstandardized coefficients	Standardized coefficients	Standard error	Significance *p*-values
Constant term	1360.533		165.765	< 0.001
Material/sintering protocol	-223.273	-.120	66.306	0.001
Layer thickness	949.520	.830	40.604	< 0.001
Thermomechanical aging	-458.438	-.245	66.306	< 0.001
R^2^	0.763			
Adjusted R^2^	0.759			
F (df = 3, 189)	201.999			

Material/sintering protocol: Zolid Gen-X/CS coded with 1, Zolid RS/HS coded with 2

Layer thickness: 0.5 mm coded with 1, 1.0 mm coded with 2, and 1.5 mm coded with 3

Thermomechanical aging: initial coded with 1 and after aging with 2

**Table 3:** Descriptive statistics for the fracture load [N] and Weibull moduli of the 4Y-TZP crowns

	Zolid RS/HS						Zolid Gen-X/CS					
	0.5 mm		1.0 mm		1.5 mm		0.5 mm		1.0 mm		1.5 mm	
	Mean (± SD)	[95% CI]	Mean (± SD)	[95% CI]	Mean (± SD)	[95% CI]	Mean (± SD)	[95% CI]	Mean (± SD)	[95% CI]	Mean (± SD)	[95% CI]
Initial												
Parametric analysis	1346 ± 42.96^a, A, β^	[1254; 1438]	2554 ± 125.7*^a, B, α^	[2286; 2823]	3120 ± 68.61^a, C, β^	[2973; 3267]	1429 ± 113.4^a, A, α^	[1186; 1671]	2634 ± 106.2^a, B, β^	[2407; 2861]	3715 ± 105.7^b, C, β^	[3489; 3941]
Weibull modulus	8.6^b, AB, β^	[5.0; 14.5]	5.8^a, A, α^	[3.3; 9.7]	13.1^a, B, α^	[7.7; 22.0]	3.5^a, A, α^	[1.9; 5.9]	7.3^a, B, α^	[4.2; 12.3]	10.4^a, B, α^	[6.1; 17.5]
After aging												
Parametric analysis	797.3 ± 157.3^a, A, α^	[461.9; 1133]	2252 ± 106.9^a, B, α^	[2024; 2480]	2683 ± 50.62^a, C, α^	[2574; 2791]	1289 ± 128.6^b, A, α^	[1014; 1564]	2087 ± 126.1^a, B, α^	[1817; 2356]	2939 ± 110.6^b, C, α^	[2703; 3175]
Weibull modulus	0.1^a, A, α^	[0.1; 0.3]	5.6^a, B, α^	[3.2; 9.3]	16.0^b, C, α^	[9.6; 26.8]	2.7^b, A, α^	[1.5; 4.6]	4.3^a, A, α^	[2.4; 7.2]	8.4^a, B, α^	[4.9; 14.2]

∗ Not normally distributed

abc Different letters present significant differences between materials/sintering protocols within one layer thickness and aging level

ABC Different letters present significant differences between layer thicknesses within one material/sintering protocol and aging level

αβγ Different letters present significant differences between aging levels within one layer thickness and material/sintering protocol

### Survival

Five Zolid RS/HS crowns with a layer thickness of 0.5 mm fractured during chewing simulation. In addition, three 0.5 mm Zolid RS/HS specimens and one 0.5 mm Gen-X/CS showed cracks in their occlusal surface after thermomechanical aging (Table 4).

**Table 4:** Fracture type distribution (*n* [95% CI]) of intact, cracked, and fractured crowns after thermomechanical aging

	Zolid RS/HS						Zolid Gen-X/CS					
	0.5 mm		1.0 mm		1.5 mm		0.5 mm		1.0 mm		1.5 mm	
	*n*	[95% CI]	*n*	[95% CI]	*n*	[95% CI]	*n*	[95% CI]	*n*	[95% CI]	*n*	[95% CI]
Intact	50.00	[23; 76]	100.00	[78; 100]	100.00	[78; 100]	93.75	[68; 100]	100.00	[78; 100]	100.00	[78; 100]
Crack	18.75	[3; 46]	0.00	[0; 21]	0.00	[0; 21]	6.25	[0; 30]	0.00	[0; 21]	0.00	[0; 21]
Fracture	31.25	[10; 59]	0.00	[0; 21]	0.00	[0; 21]	0.00	[0; 21]	0.00	[0; 21]	0.00	[0; 21]

### Fracture types and fractographic analysis

**Fig. 5:**
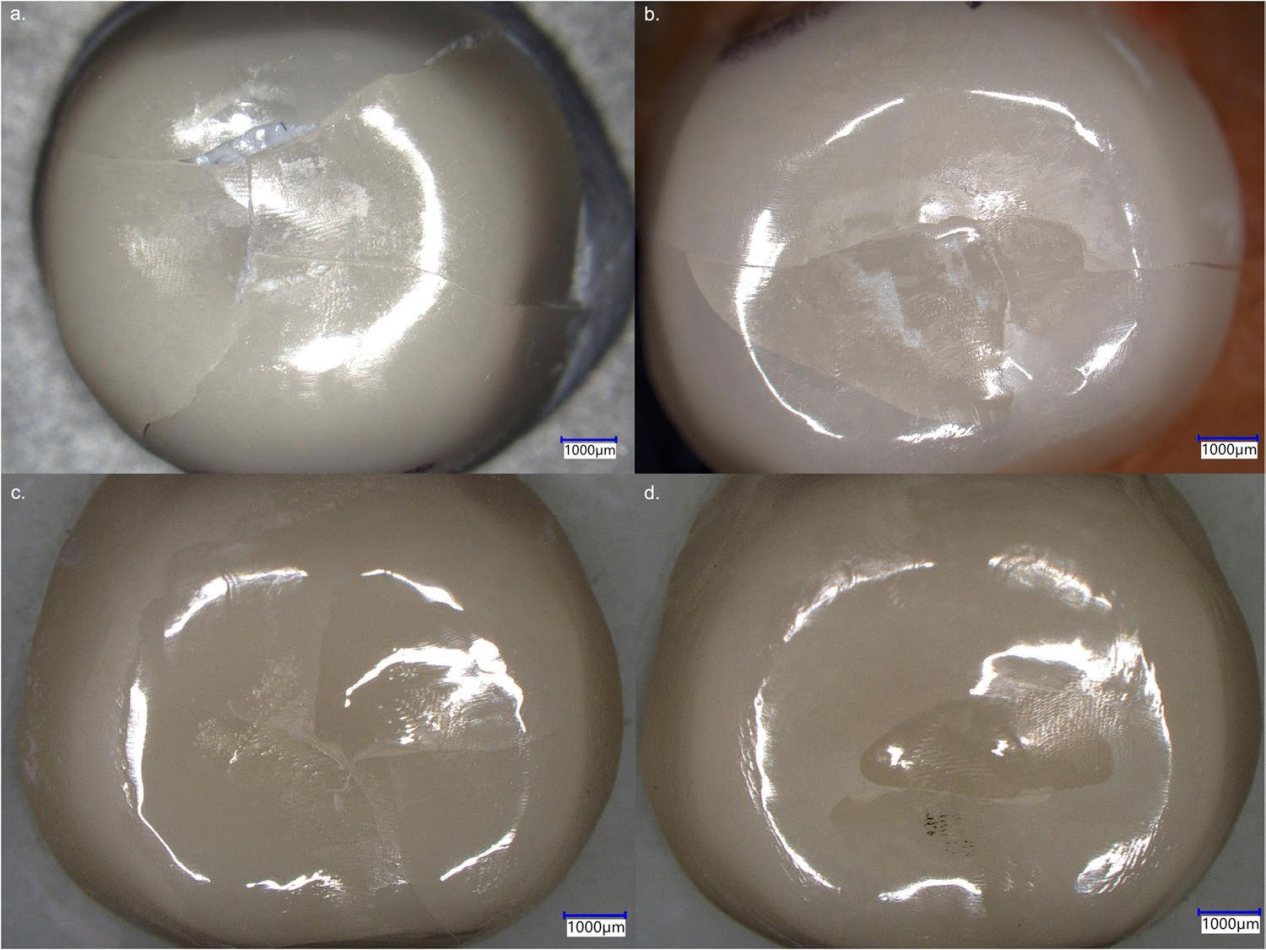
**a**–**d** View on the occlusal surface of four 0.5-mm-thick crowns (**a**–**c** Zolid RS/HS, **d** Gen-X/CS) showing typical fracture lines caused by thermomechanical aging

**Figs. 6:**
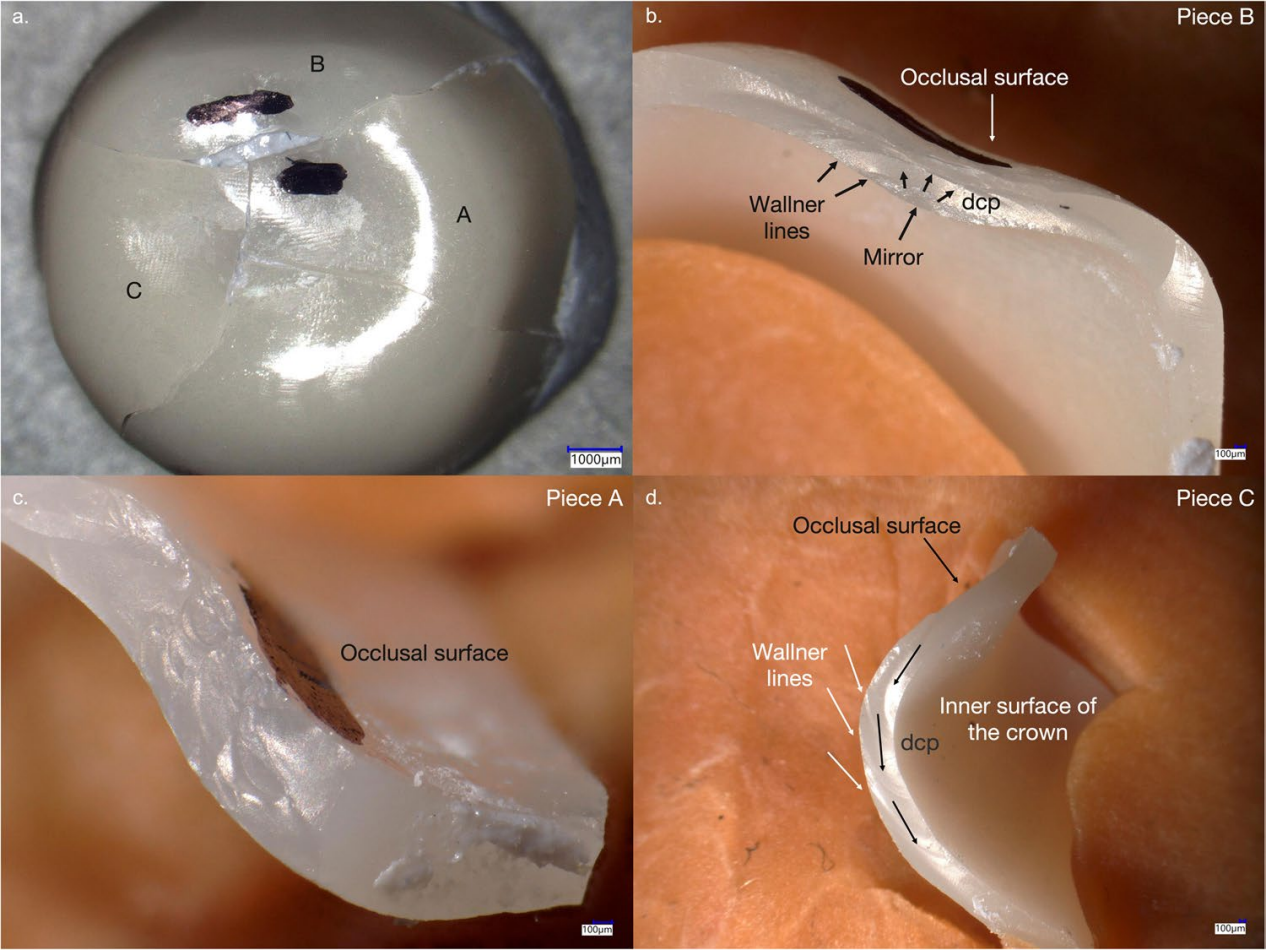
**a**–**d** Fractographic analyses of a fractured 0.5-mm-thick Zolid RS/HS specimen after thermomechanical aging (**a** occlusal view, **b**–**d** close-ups of fracture pieces A, B, and C). a. Fracture line overview from the occlusal direction, with one missing part in the center. **b** A crack, that probably initiated from a secondary fracture, running from the inside to the outside of the crown. Mirror, Wallner lines, and dcp could be identified. **c** Cyclic loading led to multiple impacts in the same area, but not exactly the same point. Wallner lines were generated by elastic impulses outside the original crack front. **d** Wallner lines indicate the dcp

**Fig. 7:**
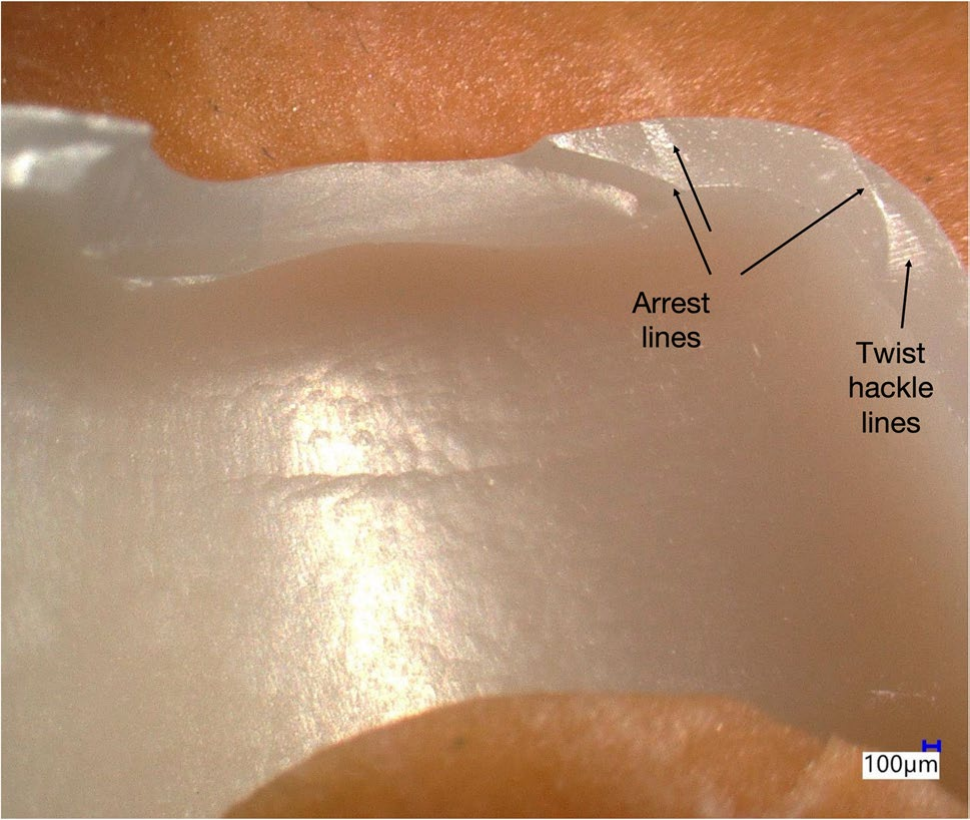
Close-up of a 0.5-mm-thick Zolid RS/HS specimen. Arrest lines caused by cyclic loading and the transient tension and stress state can be identified. Twist hackle lines leading away from the arrest line are visible, which are formed when the crack repropagates in a different direction

Cracked and fractured specimens presented the same occlusal fracture pattern. This is exemplarily shown for four different crowns in Fig. 5. In the analyzed specimens, fracture origins were generated by impacts leading to local stress by point contacts. In these cases, the impact was sufficient to create cone and radial cracks that caused crown cracks or complete crown fractures. Crack branching was also observed. Secondary fractures induced by friction and cyclic loading were visible in the form of chippings along the crack propagation. In the sliding contact area, chatter marks as typical traces for friction damage mode induced by lateral movement of the antagonist during chewing simulation were not detected. Further crack information was obtained from analyzing fracture surfaces (Figs. 6 and 7), e.g., mirrors confirming crack initiation, hackle and Wallner lines helping in identifying the direction of crack propagations, and arrest lines showing crack speed variations.

## Discussion

The aim of this investigation was to examine the influence of the material and corresponding sintering protocol, the restoration’s layer thickness, and thermomechanical aging on the 2BW and FL of 4Y-TZP crowns. The first hypothesis, that the choice of material/sintering protocol does not present an impact on the 2BW of the ceramic or respective enamel antagonist, was accepted. The second hypothesis, that neither the material/sintering protocol, nor the choice of three different layer thicknesses nor artificial aging show an impact on the fracture load of the 4Y-TZP crowns, was rejected. The high interaction between the material/sintering protocol, the layer thickness, and the aging level also affected the results. Therefore, all hypotheses were analyzed separately.

### Influence of the material/sintering protocol

As was to be expected, the two parameters volume and height loss following 2BW showed a high correlation [24, 31]. To enhance comparability to a previous investigation that examined an experimental high-speed sintering furnace [18], material loss is presented as volume loss. The material loss of the 4Y-TZP was noted to be considerably lower than that of the natural enamel antagonists. While minimum wear may in theory be desirable, the lower 2BW reported between enamel and zirconia in comparison with enamel-enamel contact points [32] may disbalance occlusion schemes and should be taken into account when planning a prosthetic restoration [33]. In contrast to earlier findings that reported high-speed sintering to result in less 2BW of 4Y-TZP than seen for a conventionally sintered control group [18], the choice of material/sintering protocol did not influence the reported material loss in the present investigation. The previous assumption that high-speed sintering may influence a zirconia’s surface structure (e.g., by inducing the formation of micro pits) and subsequently its wear behavior can thus not be confirmed [34].

For half of the tested groups, high-speed sintered Zolid RS presented lower FL values than observed for the conventionally sintered Zolid Gen-X specimens. This is in contrast to a previous investigation that reported similar or higher FL values following high-speed sintering [18]. A comparison of the absolute values between the two investigations furthermore shows the earlier findings to be considerably higher. This may be caused by using different abutment materials. While the previous investigation employed metal alloy, which has been reported to falsely increase FL values by a transfer of the applied force onto the abutment material [35], the present investigation used resin composite, a material that may deform during loading and thus result in an earlier fracture. A resin composite abutment can closely mimic the natural tooth (elastic modulus resin composite: 19 GPa, elastic modulus dentin: 18–20 GPa) and accurately reproduce a resin composite core build-up, hereby ensuring an in vitro test set-up that allows for preciser clinical deductions. Due to the substantial difference between the elastic modulus of the resin composite abutment and the zirconia restoration material (elastic modulus 4Y-TZP: ≥ 200 GPa), a resin composite abutment may even allow for a sounder elaboration of differences between groups than metal alloy abutments, that possess a similar elastic modulus to zirconia (elastic modulus metal alloy: 200 GPa). Considering the findings of the present investigation, high-speed sintering may impair a crown’s resistance to fracture. While this may be negligible for specimens of 1.5 mm layer thickness that achieved exceedingly high FL values for both sintering protocols, it is clinically relevant for aged 0.5-mm-thick specimens, where conventionally sintered Zolid Gen-X specimens achieved FL values that were about 50% higher than those of the high-speed sintered Zolid RS and thus exceeded the required maximum masticatory forces [36]. As the two 4Y-TZPs solely differ in their dyeing, the reason for this observation may lie within the employed sintering protocols. Potential causes for the lower mechanical performance of high-speed sintered specimens include an inadequate sintering of the zirconia due to a reduced holding time or an excessive grain growth caused by the higher final sintering temperature [10–12, 37]. While previous investigations have reported a similar density and microstructure for 3Y-TZP and 5Y-TZP after high-speed sintering [38, 39], investigations examining 4Y-TZP are scarce [13, 17]. While the grain size of 4Y-TZP increased with an increase in sintering temperature and led to a reduced Vickers hardness [17], biaxial flexural strength was not affected [13, 17]. The impact of the sintering parameters on the phase content of the zirconia is, as of yet, not fully understood, with an increase of the tetragonal phase and a simultaneous decrease of the monoclinic phase with a temperature increase from 1450 to 1600 °C being reported for 6Y-TZP, while a reversed trend was described for 7Y-TZP and 8Y-TZP, namely a decrease in the tetragonal and an increase in the monoclinic and cubic phase [40]. To allow a deeper understanding of the observed findings, especially with regard to the contradictory results regarding the reliability of the sintered restorations, additional investigations analyzing the grain size, phase content, and mechanical properties of a wide variety of 4Y-TZP materials in dependence on the sintering parameters are warranted.

### Influence of the layer thickness

The restorations’ layer thickness showed the highest impact on the reported FL values, with an increase in layer thickness going hand in hand with an increase in FL for both Zolid RS/HS and Gen-X/CS crowns. The increased reliability observed in conjunction with an increased layer thickness may be explained by the ability of thicker restorations to compensate existing microdefects. With five 0.5-mm-thick specimens fracturing and an additional four presenting a crack in their occlusal surface caused by the blunt impact during thermomechanical aging, 0.5-mm-thick specimens may not be able to withstand the masticatory forces observed in the oral cavity [36], despite the manufacturer indicating a minimum thickness of 0.5 mm to be suitable for manufacturing single crowns from both zirconia materials. An esthetic minimally invasive approach can thus not be implemented with Zolid RS/HS, where a minimum layer thickness of 1.0 mm should be adhered to. Further studies are warranted to investigate the performance of 0.5-mm-thick Gen-X/CS crowns more closely, as this group showed promising FL values, yet one specimen of this group showed a crack after thermomechanical aging.

### Influence of thermomechanical aging

For most groups, artificial aging showed a negative impact on the reported FL values. The thermal and mechanical stress during chewing simulation may induce microcracks that impair the crowns’ resistance to fracture and reduce the reliability of the restoration. While previous investigations reported fully stabilized zirconia materials to be resistant to aging [7, 41], 4Y-TZP may be susceptible to hydrothermal aging dependent on the doted color pigments [42]. Interestingly, 0.5-mm-thick Gen-X/CS specimens were not affected by artificial aging. This observation could hint to a successful use of this material composition and corresponding sintering protocol in minimally invasive prosthetic dentistry.

The present findings must be regarded in respect to the limitations of this in vitro investigation, which include the limited number of tested materials and sintering furnaces and the mechanical focus. Future investigations that critically examine the esthetic appearance of high-speed sintered restorations are necessary. In this context, the determination of the grain size and phase content in relation to the employed sintering protocol could enhance our understanding of the structural properties of different zirconia compositions and the resulting optical and mechanical properties. One further limitation of the present investigation is the high scattering of 2BW results, especially in respect to the 4Y-TZP crowns. This may be caused by measurement inaccuracies arising during powdering, scanning, and repositioning after chewing simulation or as a result of the inherent inhomogeneity of the employed natural enamel antagonists [43]. The high scattering observed for the 4Y-TZP crowns may also be caused by the inclusion of cracked specimens in the 2BW measurements. Future investigations should furthermore examine layer thicknesses between 0.5 and 1.0 mm, as intermediate thicknesses may provide sufficient mechanical results for manufacturing single crowns from the tested 4Y-TZPs and could thus allow a more conservative preparation of the tooth. The interpretation of the mechanical failures is limited by the lack of SEM imaging in the fractographic analysis. The lack of an a priori power analysis to determine an adequate sample size represents a further limitation of this investigation. A post-hoc power analysis that compared the fracture load of 0.5 mm Zolid RS/HS and Zolid Gen-X/CS crowns after aging showed the resulting power of a two-sided two-sample *t* test to be equal to 100% for a sample size of 16 specimens per group, an observed effect of 492 N, and a pooled standard deviation of 189 N. The choice of these two groups was based on the assumption that differences would be the least evident for aged 0.5 mm specimens that presented the lowest reliability.

While this in vitro investigation observed promising first results for the mechanical performance of high-speed sintered crowns made from esthetic multilayer 4Y-TZPs, a minimum layer thickness of 1.0 mm is recommended to ensure satisfactory long-term results.

## Conclusions

Within the limitations of this investigation, the following conclusions can be drawn:

1. The choice of material and corresponding sintering protocol did not influence the material loss during 2BW.
2. High-speed sintered Zolid RS presented similar or reduced FL values in comparison with the conventionally sintered Zolid Gen-X and should be deployed with a minimum thickness of 1.0 mm.
3. For most groups, thermomechanical aging showed a negative impact on the FL of 4Y-TZP crowns.
